# Peer Review of “Mass Testing With Contact Tracing Compared to Test and Trace for the Effective Suppression of COVID-19 in the United Kingdom: Systematic Review”

**DOI:** 10.2196/28719

**Published:** 2021-04-12

**Authors:** Archisman Roy

**Affiliations:** 1 Department of Physics Faculty of Mathematical Science Institute of Sciences, Banaras Hindu University Asansol India

**Keywords:** COVID-19, SARS-CoV-2, test and trace, universal testing, mass testing, contact tracing, infection surveillance, prevention and control, review

*This is a peer-review report submitted for the paper “Mass Testing With Contact Tracing Compared to Test and Trace for the Effective Suppression of COVID-19 in the United Kingdom: Systematic Review.”*

## Round 1 Review

### General Comments

The paper titled, “Mass Testing With Contact Tracing Compared to Test and Trace for the Effective Suppression of COVID-19 in the United Kingdom: Systematic Review” [1] is well textured and finely written with sufficient subpoints and clear divisions. The English used is simple but lucid enough and enriched compared to an international journal standard. From the very beginning, the title is so effective and descriptive that it provides a brief outline for the readers. The abstract is finely written, pointing to the outcomes of the paper, which also includes an applaudable inculcation of a nutshell overview of the methods in use. The data analysis, results, and discussion, as well as the detailed structure of the major findings, *P* values, statistical coefficients, and so on, conform to the author's guide. But there are a few minor typographical errors that need to be checked to improve the write-up. Please consider the points in the *Minor Comments* section. The citations mentioned in the paper fit well with the context. Overall, the description of the content is very clear, and every point is academically backed up with either derivations or scientifically validated information, which is commendable. The figures (mainly the flow chart) are very precise but wonderfully narrative. The *Methods* section has been presented in good harmony with the objectives, outcomes, and strategy although my view on the outcomes differs a bit (please look at the *Specific Comments* section). The data analysis section is well structured, maintaining the flow of data management. In total, the paper is a worthy piece but, in my view, it may require a few minor changes. Kindly refer to the following comments.

### Specific Comments

1. It is crucial to remove a few points to make the paper easily acceptable for readers and better its viability. I suggest cutting a bit in the *Research in Context* section. It is fine to have a short review of the literature, but the paper overall is full of it so shortening the aforesaid section may increase its impact.

2. I also do not find the validity of having the *Definitions* section. When an author is proposing a new theory bearing some new terminology, this section is needed, but getting acquainted with formal terms is the prime duty of readers.

3. In the *Data Extraction* section, you paced on the author’s details, specifically its singularity, which seems inapplicable to me. Please consider jotting that area down again in a twisted fashion.

It is really commendable the way you have composed this paper. As mentioned earlier, the writing style is very soothing and effective as a worthy academic contribution. Still, a few points need more attention, which have been further segmented into major and minor comments.

### Major Comments

1. Reviewers are not asked to look into the grammar and spelling very thoroughly, so I am giving an overview. Please consider reading the paper again as a few words seems to mismatch their application. For instance, in the following sentence in the *Background* section, “I concur with the...,” the word “concurs” is out of context, so please look into the matter.

2. In the *Research to Context* prior to *Study* section, there is mention of a review; please cite it for a better scholarly approach.

3. The paper bears a good philosophical measure of uncertainty introduced. This section is very nicely formatted in an appreciable way.

4. Outcomes occur within the *Methods* section, which is not advised. Adding the outcomes here creates a sense of biasedness since outcomes can never be assumed beforehand, which is why you may consider removing these points from here.

5. The section *How the Intervention Should Work* ought to be included under *Methods*. I would suggest replacing its name with *Active Runs of Intervention* as a subsection. The objectives and outcomes further include some basic information about COVID-19 and the strain itself. This is really unimportant, so please remove that portion to reduce the word count of the paper.

6. In the *Outcomes* section, the third point: there is a point on safety; however, the tone of safety in mass testing methods seems to be understated so I would like to propose emphasizing the safe nature of MTT. Again, the paper is really a worthy piece for me, so consider these points as a proposal for improvement.

### Minor Comments

1. Many paragraphs lack the use of full stops at the end line. Please have it checked with a little care.

2. There are some issues with grammatical usages; please consider fixing them. You may opt for artificial intelligence–based screening to get better results (eg, Grammarly).

3. The importance of the separate column for vote count in the relative study of TT and MTT is not clear to me so please try to express its viability in a line or so for clarity.
